# New Insights into the Mechanism of Insulin-like Peptide 3 (*INSL3*) Regulating the Growth and Development of *Bovine* Follicular Granulosa Cells

**DOI:** 10.3390/ijms27010405

**Published:** 2025-12-30

**Authors:** Hongxian Li, Fenglou He, Xinye Li, Junjie Nie, Hasnain Ali Khan, Chao Chen, Jinling Hua

**Affiliations:** College of Animal Science, Anhui Science and Technology University, Fengyang 233100, China; lihongxian20221@outlook.com (H.L.);

**Keywords:** *Dabie Mountain cattle*, *INSL3*, follicular development, granulosa cells, proliferation

## Abstract

*Dabie Mountain cattle* are characterized by their ability to tolerate coarse feed, strong disease resistance, and delicious meat. Lower reproductive efficiency has become one of the key factors limiting its development. Therefore, this study investigated the developmental patterns of *Dabie Mountain cattle* follicles and screened key candidate genes for in vitro experimental validation. Research collected granulosa cells from small follicles (<5 mm), medium (5–8 mm), and big (>8 mm), followed by RNA extraction for transcriptomic sequencing. A total of 20,775 genes were identified, including 13,777 (66.3%) differentially expressed genes (DEGs). DEGs showing up-regulation and down-regulated in B vs. S, B vs. M, and M vs. S groups were collected. A total of 19 commonly up-regulated DEGs across the three groups were identified, including genes such as *DEFB*, *FAM124A*, and *RASSF10*. Additionally, 227 commonly down-regulated DEGs were identified, including genes such as *INSL3*, *GAS7*, and *PAQR7*. Protein interaction network analysis revealed an interaction between *INSL3* and *STAR*. *Bovine* ovarian granulosa cells (GCs) were collected to investigate the effect of the *INSL3* on GCs proliferation. The results revealed that *INSL3* expression was highest in small follicles and was almost absent in big follicles. Subsequently, the *INLS3* gene was knocked down in GCs using small interfering RNA. RT-qPCR results demonstrated that both si-INSL3 (239) and si-INSL3 (392) significantly knock down *INSL3* expression (*p* < 0.01), si-INSL3 (239) for follow-up research. CCK-8 was used to assess cell proliferation, revealing that *INSL3* knockdown significantly enhanced GCs viability and number at 24, 48, and 72 h (*p* < 0.05). Flow cytometry was used to detect cell cycle distribution. The results showed that knockdown of *INSL3* expression significantly decreased the proportion of G1 phase cells and significantly increased the number of S phase cells (*p* < 0.01). RT-qPCR was used to detect the expression of cell proliferation-related genes. The results showed that compared with the siNC group, the expression levels of *Myc*, *PCNA*, *Cytochrome C*, and *Cyclin D1* were significantly increased in the si-INSL3 group. In conclusion, knockdown of *INSL3* affects follicular development in *Dabie Mountain cattle* by regulating granulosa cell proliferation in the ovaries, providing new insights into the regulatory mechanisms of follicular development in cattle.

## 1. Introduction

*Dabie Mountain cattle* is one of the unique local yellow cattle breeds in China, belonging to the dual-purpose and meat use type of yellow cattle breeds. Under suitable feeding conditions, high-quality snowflake beef can be produced, which has the characteristics of tolerance to extensive feeding, strong disease resistance, and delicious meat [1]. But its reproductive rate is relatively low, with cows starting to heat at an average age of 1.5 and a gestation period of about 274 days. Breeding activities are mostly concentrated from May to July, and bulls only have mating ability after the age of 2.5. The existing inventory is less than 2000 heads and is showing a downward trend year by year [2]. However, the reproductive rate is influenced by various factors and complex regulatory mechanisms both within and outside the organism [3,4], particularly the developmental stage of ovarian follicles in vivo [5,6]. Therefore, elucidating the regulatory mechanisms underlying *Dabie Mountain cattle* follicular development is crucial for enhancing reproductive efficiency.

As is well known, the ovaries are the reproductive glands of female animals, serving as both a source of germ cells and a major donor of steroid hormones. Follicles are mainly composed of oocytes, granulosa cells, and theca cells, and are the basic units for mammalian ovarian function. Follicles in female animals at different developmental stages have different physiological functions and morphological structures. Its development process presents the characteristics of follicular waves, which is a continuous dynamic process mainly including three stages of recruitment, selection, and dominance, and finally enters the ovulation stage. Relevant studies showed that when the diameter of follicles reached 8 mm, the differentiation of follicles began to shift. Specifically, except for dominant follicles, the growth and development of other follicles became slow [7]. At this time, this follicle is defined as the dominant follicle, while other follicles are defined as subordinate follicles [8,9,10]. The transcriptome sequencing analysis of follicles (follicle diameter > 10 mm) after differentiation showed that follicles at this stage were involved in cell mitosis, DNA replication, and gene expression of cell structure and repair [11]. At the same time, *mgarp*, *GLDC*, *chst8*, *GPx3* and other genes were found in the detection of small follicles (3–5 mm) and big follicles (>9 mm), which were considered as potential markers of GC [12]. Circular RNAs were detected in granulosa cells of 5.0–6.9 mm *porcine* follicles to participate in follicular development through a variety of mechanisms, including regulating the dynamic balance of follicular growth through competitive binding miRNAs, interacting with RNA binding proteins, regulating gene splicing and translation, and regulating follicular endocrine function [13]. Although there are many studies on different stages of follicular development, and many differentially expressed genes and related biological functions in different follicular diameters have been identified, the exploration of different follicular development is still imperative.

*INSL3* (Insulin-Like Peptide 3), also known as Leydig insulin-like hormone (Leydig *LH*), is an important member of the insulin/relaxin family. This peptide hormone was first discovered in Leydig cells of rodent testes in the 1990s [14,15]. Similar to members of the insulin and *INSL5* families, *INSL3* is a small molecule secreted peptide hormone that primarily exerts its biological effects by binding to its specific receptor *RXFP2* (Relaxin Family Peptide Receptor 2) [16]. Initially, *INSL3* was considered a key endocrine factor in embryonic testicular descent, but recent studies have shown that its function is far from limited to reproductive development, and it is involved in reproductive regulation, bone metabolism, energy homeostasis, and various endocrine processes in adult males and females [17]. For example, during male fetal development, *INSL3* mainly regulates the second stage of testicular descent (inguinal scrotal stage); in adult males, it mainly plays a role in maintaining Leydig cell homeostasis and reproductive function, while in adult females, it plays a biological role in regulating follicular development, luteal function, and ovarian endocrine function [18,19]. In addition, relevant research reports suggest that *INSL3* has potential roles in bones, muscles, metabolism, and the immune system [20,21]. With the improvement of high-precision measurement technology and the deepening of *RXFP2* signaling mechanism research, *INSL3* is expected to become an important clinical biomarker for evaluating reproductive health, bone health, and metabolic status, and may develop into a new therapeutic target [18,22].

## 2. Results

### 2.1. Analysis of Transcriptomes of Follicular Granulosa Cells from Different Follicles

**Figure 1:**
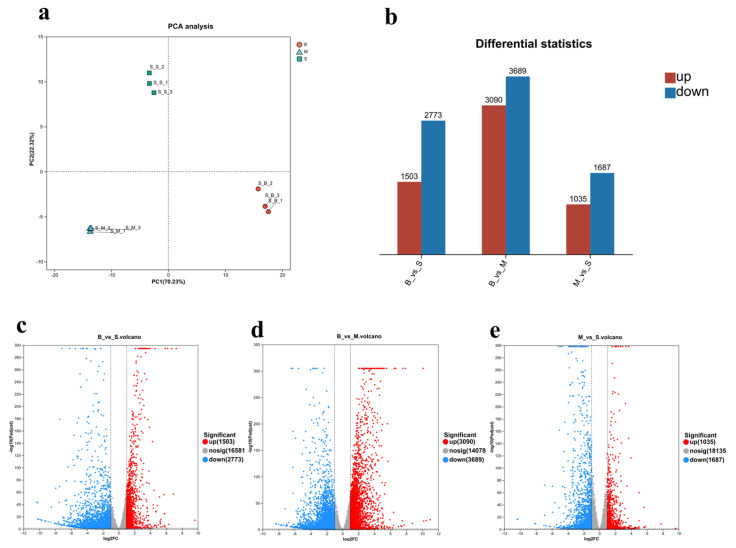
Differentially Expressed Genes in Follicles at Different Developmental Stages. (**a**): PCA plot grouped by B vs. S, B vs. M, and M vs. S follicle stages. Each stage is represented by its own color; (**b**): The number of DEGs in the three groups; (**c**–**e**): representing heatmaps of differentially expressed genes (DEGs) showing upregulation (red) and downregulation (blue) in B vs. S, B vs. M, and M vs. S, respectively.

To investigate the regulatory mechanisms associated with *Dabie Mountain cattle* follicular development, this study collected granulosa cells from follicles of different sizes (<5 mm, 5–8 mm, and >8 mm) and performed transcriptome sequencing. The results of the principal component analysis (PCA) indicate no significant separation was observed among the three sample groups, indicating strong reproducibility across samples and providing a reliable foundation for subsequent analyses (Figure 1a). Differential expression gene (DEGs) analysis reveale among the 20,775 identified genes, 13,777 (66.3%) were differentially expressed (Figure 1b). Compared to Group S, Group B identified a total of 4276 DEGs (1503 up-regulated and 2773 down-regulated) (Figure 1c); Compared to Group M, Group B identified a total of 6779 DEGs (3090 up-regulated and 3689 down-regulated) (Figure 1d); Compared to Group S, Group M identified a total of 2722 DEGs (1035 up-regulated and 1687 down-regulated) (Figure 1e).

### 2.2. Selection of Crucial Genes Regulating Follicular Development

**Figure 2:**
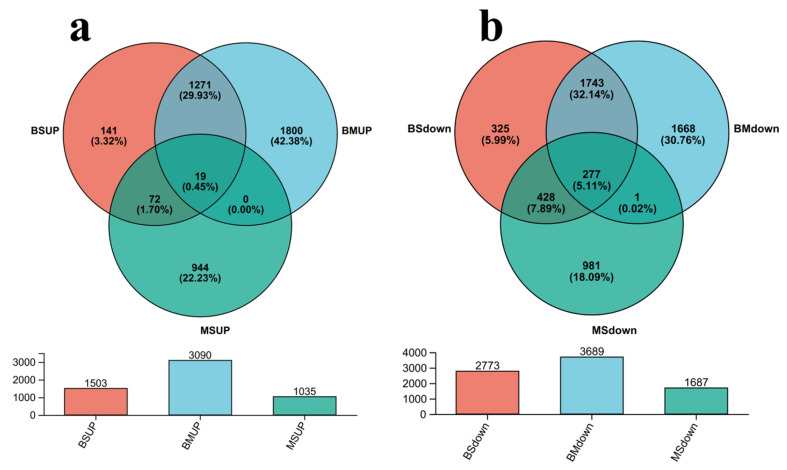
Follicular Development Critical Regulatory Genes. (**a**): Venn analysis results of BSUP (Compared with group S, the number of up-regulated DEGs in group B), BMUP (Compared with group M, the number of up-regulated DEGs in group B), and MSUP (Compared with group S, the number of up-regulated DEGs in group M); (**b**): Venn analysis results for BSdown (Compared with group S, the number of down-regulated DEGs in group B), BMdown (Compared with group M, the number of down-regulated DEGs in group B), and MSdown (Compared with group S, the number of down-regulated DEGs in group M).

To identify key candidate genes regulating follicular development, this study screened all up and down-regulated genes across the B vs. S, B vs. M, and M vs. S groups. Based on the aforementioned analysis, the up-regulated genes and down-regulated genes established distinct gene sets for Venn analysis, resulting in the identification of 19 up-regulated DEGs (Figure 2a), including genes such as *DEFB*, *FAM124A*, *RASSF10*. Additionally, the identification revealed 277 down-regulated DEGs (Figure 2b), including genes such as *DAZL*, *GAS7*, *INSL3*, and *PAQR7*.

### 2.3. Gene Ontology (GO) and Kyoto Encyclopedia of Genes and Genomes (KEGG) Analysis

**Figure 3:**
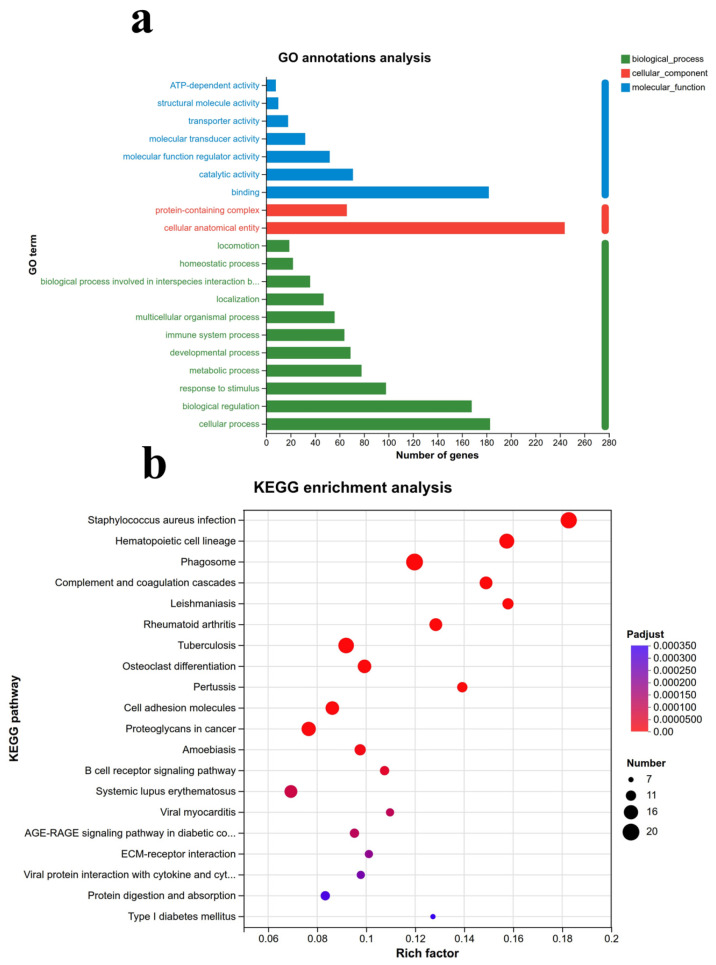
GO and KEGG analysis. (**a**) GO functional enrichment analysis of DEGs; (**b**) KEGG enrichment analysis of DEGs.

The Fisher test was employed to conduct functional enrichment analysis of the co-downregulated and co-upregulated gene sets mentioned above. The results indicated that, at the biological process (BP) level, these gene sets were primarily involved in cellular processes, biological regulation, responses to stimuli, metabolic processes, developmental processes, and immune system processes, with the most significant number of genes enriched in cellular processes. At the cellular component (CC) level, they were predominantly associated with cellular anatomical entities and protein-containing complexes. At the molecular function (MF) level, the gene sets were mainly involved in binding, catalytic activity, molecular function regulation, molecular transduction, transporter activity, structural molecular activity, and ATP-dependent activity, with the binding function genes being the most abundant (Figure 3a).

KEGG enrichment analysis showed that the gene set was mainly enriched in the relaxin signaling pathway, ECM receptor interaction, AGE-RAGE signaling pathway, B cell receptor signaling pathway, cell adhesion molecules, coagulation cascade, and other complex signaling pathways in diabetes complications (Figure 3b).

### 2.4. Protein–Protein Interaction (PPI) and Hub Genes

**Figure 4:**
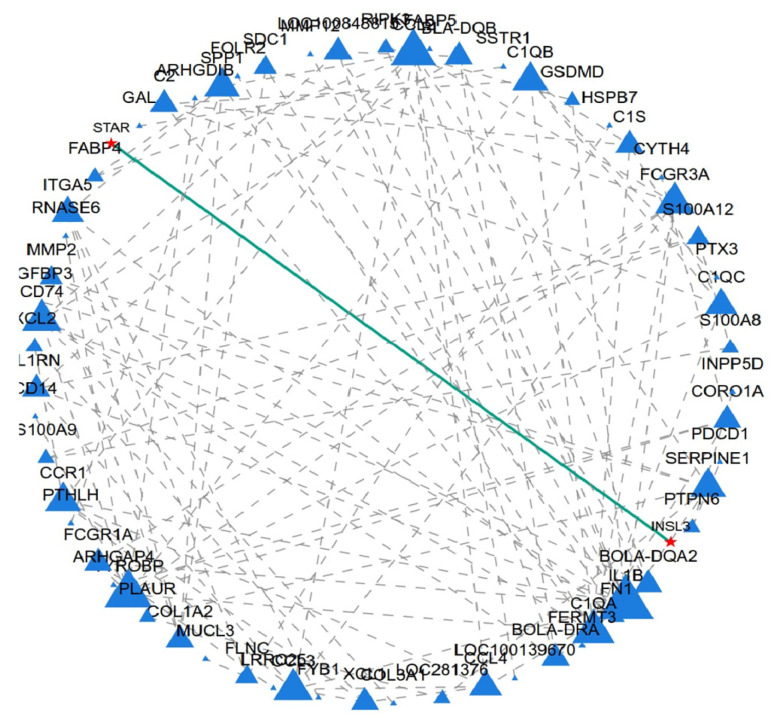
Protein interaction analysis results of 296 differentially expressed genes.

Through protein interaction network analysis, it was found that there is an interaction relationship between *INSL3* and *STAR* (Figure 4). However, the specific mechanism behind this relationship is still unclear. *STAR*, as a key regulatory protein in gonadal steroid hormone synthesis, indicates that *INSL3* may also play an important role in follicular development in female animals. These novel findings could challenge previous research on *INSL3* and may serve as a new target marker for the treatment of human ovarian diseases and for enhancing reproductive rates in ruminants.

### 2.5. Detection of INSL3 Expression in Follicles at Different Developmental Stages and Interference Efficiency

**Figure 5:**
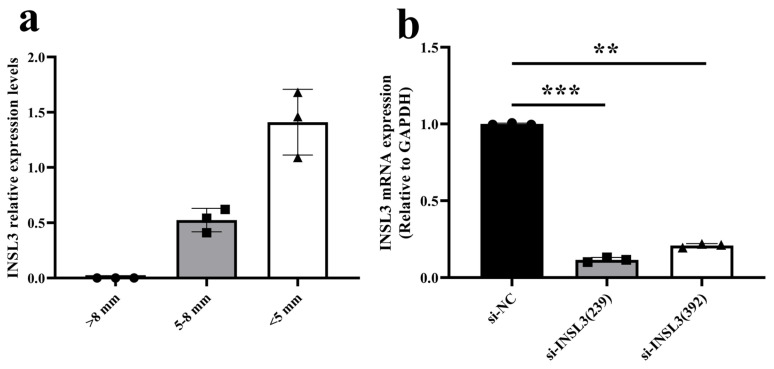
Expression of *INSL3* and detection of intervention efficiency. (**a**) Expression of *INSL3* in GCS with different follicular diameters. (**b**) *INSL3* mRNA expression was successfully decreased in *Dabie Mountain cattle* GCs transfected with si-INSL3 (20 μM). mRNA levels by RT-qPCR relative to endogenous control *GAPDH*. The results are indicated as means ± SEM of three independent experiments. ** *p*< 0.01, *** *p* < 0.001.

GCs were collected to investigate the effect of the *INSL3* on GCs proliferation. The results revealed that *INSL3* expression was highest in small follicles and was almost absent in big follicles (Figure 5a). The ovarian granulosa cells of *Dabie Mountain cattle* were grouped and transfected with *INSL3* siRNA (si-INSL3 (239, 392)) and a negative control (si-NC), respectively. RNA was extracted 48 h later, and the interference efficiency of si-INSL3 was detected using the real-time fluorescence quantitative PCR method. The results are shown in Figure 5b, compared with the control group, the relative expression levels of si-INSL3 (239) and si-INSL3 (392) mRNA were significantly downregulated by 88% (*p* < 0.001) and 79% (*p* < 0.01), respectively. This research selected si-INSL3 (239) for subsequent experiments because it was the most effective siRNA.

### 2.6. Knockdown of INSL3 Affects Granulosa Cell Proliferation and Cell Cycle Progression

**Figure 6:**
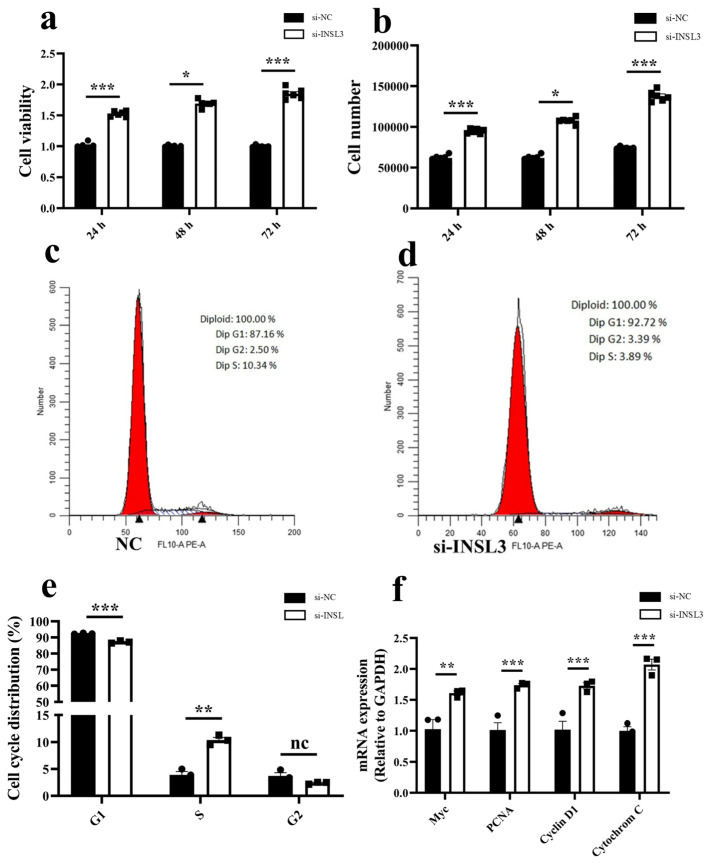
Knockdown of *INSL3* expression affects GCs proliferation, cycle, and proliferation-related genes. (**a**,**b**) CCK-8 kit was used to evaluate the cell vitality and cell number of GC transfected with si-INSL3 and siNC at 24, 48 and 72 h, respectively. (**c**–**e**) *Dabie Mountain cattle* GC was transfected with si-INSL3 and incubated for 48 h before being saturated in PI, and the FACS and GC cycle were determined. (**f**) Expression levels of *Myc*, *PCNA*, *Cytochrome C*, and *Cyclin D1* genes in GC transfected with siRNA and siNC were determined by RT-qPCR against endogenous control *GAPDH*. All results are presented as means ± SEM. Significance difference, * *p* < 0.05, ** *p* < 0.01, *** *p* < 0.001.

To investigate the effect of *INSL3* on granulosa cell proliferation, cell proliferation was assessed using the CCK-8 assay. Results indicated that following *INSL3* gene RNA interference treatment, the cell vitality and cell number of *bovine* follicular granulosa cells were significantly increased compared to the si-NC group (*p* < 0.01) (Figure 6a,b). Flow cytometry analysis revealed that compared with the control group, the si-INSL3 group exhibited a significant downregulation in the G1 phase and a significant up-regulation in the S phase (*p* < 0.01), while no significant difference was observed in the G2 phase (*p* > 0.01) (Figure 6c–e). Finally, to investigate the molecular mechanisms underlying *INSL3* regulation of GCs proliferation, RT-qPCR analysis revealed significantly elevated relative expression levels of the cell proliferation-related genes *Myc*, *PCNA*, *Cytochrome C*, and *Cyclin D1* (*p* < 0.01) compared to the control group (Figure 6f). These findings suggest that *INSL3* may function as a novel growth-inhibitory factor in *bovine* follicular granulosa cells, indicating its involvement in a series of complex bio-logical processes and providing a theoretical basis for further elucidation of its molecular mechanisms.

## 3. Discussion

Currently, there are many studies on the different developmental stages of ovarian follicles in female animals, and these studies have elucidated the dynamic biological processes behind follicular changes and their associated molecular functional mechanisms. However, the follicular development process in the ovaries of female animals is very complex, especially the regulatory mechanisms of granulosa cells. In studies of *bovine* oocytes with diameters of 60–120 µm, it was found that during the growth phase of *bovine* oocytes, the role of oxidative phosphorylation diminishes and is accompanied by an increase in the level of expression of maternal genes and transcriptional regulators, and that the level of expression of about 5000 genes remains unchanged. When the diameter of the oocyte exceeded 100 μm, the expression of genes associated with cytoplasmic activity was replaced by genes associated with nuclear activity, such as chromosome segregation [23]. Additionally, relevant studies have explored the developmental patterns and regulatory mechanisms of follicles across different species. Transcriptome sequencing analysis of granulosa cells from 3–5 mm diameter pig follicles identified a big number of differentially expressed genes (DEGs). Among these, 51 DEGs were implicated in steroid biosynthesis, cell adhesion molecules, and TGF-β signaling pathways, Genes such as *LHR*, *ACACB*, and *CXCR4* were identified as key candidate genes through interaction network analysis [24]. In the transcriptomes of chicken follicular granulosa cells at 4–8 mm, 9–12 mm, and 40 mm stages, 41 differentially expressed genes were identified that are closely associated with the reproductive process. These include *UTRN*, *LOC422926*, *MYH11*, *CD9*, *TIGAR*, *RARA*, *PLBD1*, *PPP1CB*, *RMDN2*, *SORCS2*, *SLC9A5*, *PDLIM4*, and *PPP4R4*. Furthermore, *RMND2* expression showed negative correlations with differentiation markers *FSHR* and *AMH*, as well as proliferation marker *CDK2*, and positive correlations with steroidogenesis markers *CYP11A1*, *STAR*, and *ESR1* [25]. Research indicates that alterations in carbohydrate metabolism within the cumulus-oocyte complex may lead to reduced oocyte fertilization capacity in both small follicles (≤11.7 mm) and big follicles (≥12.7 mm) exposed to exogenous GnRH-induced gonadotropin surges [26]. As one of the endemic yellow cattle breeds in China, *Dabie Mountain cattle* are characterized by roughage tolerance and delicious meat quality, but low reproductive rate. In order to analyze the follicular development and regulatory mechanism of *Dabie Mountain cattle*, this research collected granulosa cells from small follicles (<5 mm), medium follicles (5–8 mm), and large follicles (>8 mm) of *Dabie Mountain cattle* for transcriptome sequencing, and identified a total of 20,775 genes, of which 13,777 were differentially expressed (DEGs). This further confirms the existence of a highly coordinated gene regulatory network among granulosa cells during follicular development, collectively governing proliferation, differentiation, and steroidogenesis [16,27,28,29]. Principal component analysis (PCA) revealed significant transcriptome segregation between small, medium and large follicle groups, further validating the reliability of the transcriptome dataset [30]. GO functional enrichment analysis revealed that GEGs may participate in extracellular matrix receptor interactions, and the extracellular matrix plays a crucial role in oocyte maturation [31,32,33,34]. In addition, DEGs were found to be enriched in the AGE-RAGE signaling pathway in KEGG enrichment analysis, suggesting that DEGs may indirectly affect follicle development through activation of the AGE-RAGE signaling pathway, a finding that further confirms previous evidence that advanced glycosylation end-products (AGEs) impair ovarian function and follicle quality [35,36,37]. Meanwhile, the enrichment of DEGs in relaxin-related signaling pathways, cell adhesion molecules, and the coagulation cascade suggests that the ovarian microenvironment plays an important regulatory function by modulating these pathways [38,39].

This study identified a total of 227 downregulated genes, among which *INSL3* exhibited the highest relative expression in small follicles and was nearly absent in big follicles. Transcriptome analysis revealed that insulin-like growth factor-3 (*INSL3*) regulates the biological functions of *bovine* follicular granulosa cells at different developmental stages, especially in follicles less than 5 mm and 5–8 mm in diameter. *INSL3* is mainly secreted by testicular mesenchymal stromal cells, and acts as a paracrine factor to regulate follicular growth through its receptor *RXFP2* [40]. Furthermore, cross-species studies have also demonstrated the biological function of *INSL3* in the ovaries [16,41,42,43,44]. These results suggest that follicles at different developmental stages affect *INSL3* levels, which indirectly affects follicular growth and development. To investigate the regulatory role of this gene in follicular development in *Dabie Mountain cattle*, ovarian granulosa cells were used as an in vitro model to assess its effects on cell proliferation and the cell cycle. First, protein-protein interaction (PPI) network analysis revealed a potential association between insulin-like growth factor 3 (*INSL3*) and steroidogenic acute response protein (*STAR*), which not only mediates cholesterol transport to mitochondria but also directly regulates steroidogenesis [45,46,47], a phenomenon that implies that *INSL3* may indirectly regulate steroidogenesis through modulating the this phenomenon implies that *INSL3* may indirectly play an important role in the regulation of steroidogenesis by modulating the function of *STAR*. Increasing evidence suggests that microRNAs act with the nuclear receptors *NR5A1* and *NR4A1* to regulate *STAR* and *INSL3* expression [48,49]. These studies reveal that the *INSL3*-*STAR* regulatory axis may play a potential biological function during follicular development. Functional validation by RNA interference demonstrated that knockdown of *INSL3* significantly increased granulosa cell vitality and up-regulated the expression of genes associated with proliferation and cell cycle progression, including *Myc*, *PCNA*, *Cytochrome C*, and *Cyclin D1*. Notably, the proliferation markers *Myc* and *PCNA* were strongly associated with granulosa cell proliferation [50,51,52,53]. In studies linking *INSL3* signaling disorders to the follicular cell survival pathway, increased *Cytochrome C* expression is a hallmark feature of mitochondria-mediated cell apoptosis [54]. Flow cytometry analysis showed that *INSL3* knockdown resulted in a significant decrease in G1 phase and a significant increase in S phase, while no significant changes were seen in G2 phase compared to controls. Furthermore, the results of *INSL3* knockdown experiments and studies using RNA interference (RNAi) targeting other hormones or receptors collectively validate the reliability of RNAi technology in follicular function research [55,56,57,58]. Based on the above circumstances, this research findings closely correlate with previous experimental evidence regarding *INSL3* regulation of follicular development.

To sum it up, the present study suggests that insulin-like growth factor-3 (*INSL3*) may inhibit the proliferation of *bovine* follicular granulosa cells. This suggests that it may synergize with other factors to regulate follicular development through specific signaling pathways. Future studies should utilize in vivo models to systematically analyze the *INSL3*-*STAR* regulatory axis, providing a theoretical basis for the targeted treatment of ovarian diseases and the enhancement of reproductive efficiency in livestock and poultry.

## 4. Materials and Methods

### 4.1. Animals and Experimental Design

**Figure 7:**
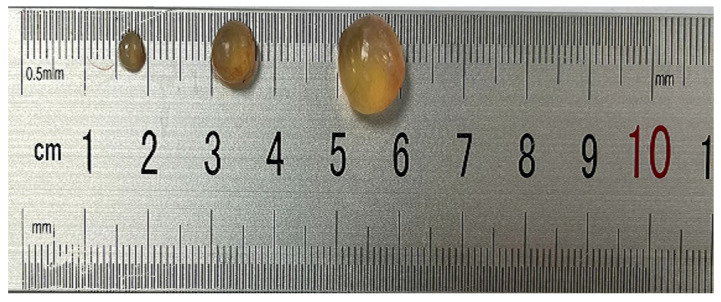
Follicle diameter size.

The samples were collected from the slaughterhouse of Wanjia Group in Taihu County, Anqing City, Anhui Province. Ovarian tissue was collected from both sides of the uterus using sterile surgical scissors and immediately washed 3–4 times with 75% alcohol and 37 °C PBS solution, then transferred back to the laboratory in a water cup containing 37 °C penicillin-streptomycin PBS buffer. Upon arrival, the samples were washed 2–3 times with 75% alcohol and a 37 °C PBS buffer solution, and then transferred to a clean workbench for follicular fluid extraction. Based on the diameter of the follicles, they were categorized into three groups: small follicles (S, <5 mm), medium follicles (M, 5–8 mm), and big follicles (B, >8 mm) (Figure 7). When extracting follicular fluid, sterile and enzyme-free 5 mL syringes were used to extract follicular fluid from each group (*n* = 3). Filter the extracted follicular fluid from each group through a 40 nm cell sieve, then transfer it to a 1.5 mL sterile enzyme-free centrifuge tube and centrifuge at 1200 rpm for 5 to 8 min. Discard the supernatant to obtain the cell pellet.

This study has been approved by the Experimental Animal Ethics Committee of Anhui Science and Technology University, with approval number [2025092]. Subsequently, the obtained cells were cultured in DMEM medium supplemented with 10% fetal *bovine* serum and 1% penicillin-streptomycin at 37 °C under 5% CO_2_ and saturated humidity conditions for subsequent studies.

### 4.2. RNA Extraction, Library Preparation, and Sequencing

According to the manufacturer’s protocol (Invitrogen, Waltham, MA, USA), TRIzol^®^ reagent was utilized to extract total RNA from each follicle group. The quality of RNA was assessed using a 5300 Bioanalyzer (Agilent 5300) and quantified with an ND-2000 (NanoDrop 2000 Technologies). Only high-quality RNA samples (OD260/280 = 1.8–2.2, OD260/230 ≥ 2.0, RQN ≥ 6.5, 28S:18S ≥ 1.0, >1 μg) were employed to construct the sequencing library. According to the manufacturer’s recommendations, an mRNA library was constructed using the Illumina Stranded Total RNA Prep method and Ribo Zero Plus. The sequencing library was constructed on the NovaSeq X Plus platform at Majorbio Bio-Pharm Technology Co., Ltd. (Shanghai, China) utilizing the NovaSeq Reagent Kit.

### 4.3. Protein–Protein Interaction (PPI) and Hub Genes

Protein interaction network analysis was performed on 296 DEGs. After the construction of the protein interaction network, Network X 2.8 in Python 3.7 was used for the visualization of the gene network. To pinpoint the principal crucial nodes of the interaction network, various topological parameters like degree, betweenness, compactness, clustering coefficient, etc., were computed. The top 20 differentially expressed genes (DEGs) highlighted by each method were selected, and through intersection analysis, DEGs identified by at least five methods were classified as Hub DEGs.

### 4.4. Dabie Mountain Cattle GCs Isolation and Culture

Ovaries from non-pregnant *Dabie Mountain cattle* were collected from slaughterhouses. Ovaries exhibiting luteal cysts or follicular cysts were visually identified and discarded. Follicles were dissected using a scalpel and categorized into three size groups based on diameter: <5 mm, 5–8 mm, and >8 mm. Follicles of appropriate size were selected for subsequent experiments. Collect follicular fluid using a syringe with a 10-gauge needle. Transfer the fluid to a 50 mL centrifuge tube fitted with a 40 nm cell strainer. After filtration, aliquot the follicular fluid into 2 mL EP, Centrifuge at 1200 rpm for 5 min and discard the supernatant. Resuspend the pellet in DMEM, centrifuge at 1200 rpm for 3 min, and collect the cell pellet. Repeat this step 2–3 times to obtain relatively pure Dabao Mountain cattle granulosa cells. Place granulosa cells in DMEM medium supplemented with 10% fetal *bovine* serum and 1% penicillin/streptomycin (Hyclone). Culture in a 6-well plate at 37 °C with 5% CO_2_ for 48 h. Change the medium every 24 h during culture.

### 4.5. INSL3 siRNA Transfection

According to the known gene sequences of cattle in the GenBank database using Primer Premier 5.0 software, primers were designed for the *INSL3* gene, cell cycle-related protein gene *Cyclin D1* and *Cytochrome C*, cell proliferation-related protein genes *Myc* and *PCNA*, and internal reference gene *GAPDH*. Both siRNA and NC-siRNA (without any target sequence) were purchased from Suzhou Jima Gene Co., Ltd. (Suzhou, China). The primers were synthesized by Beijing Qingke Biotechnology Co., Ltd. (Nanjing, China). The cells were transfected with 20 nM of siRNA compared to NC using Lipofectamine RNAiMAX reagent in Opti-MEM medium (Life Technology, Inc., Carlsbad, CA, USA) instructions. Cells were harvested 48 h after transfection for mRNA expression. Small RNA interference sequences specific to *INSL3* (si-INSL3) used in this study are given in Table 1.

### 4.6. RNA Extraction and Reverse-Transcription Polymerase Chain Reaction (RT-PCR)

Cells were lysed 48 h post-transfection. Total RNA was extracted using a kit (RNApure Fast Tissue & Cell Kit; Kangwei Century Biotechnology Co., Ltd., Taizhou, China), followed by subsequent cDNA synthesis according to the manufacturer’s instructions. RNA quality and quantity were assessed by spectrophotometry (Nanodrop 2000 analyzer; Thermo Scientific, Wilmington, DE, USA). Finally, 1 µg of qualified total RNA (260/280 ratio 1.8–2.1) was reverse transcribed using a kit (HiFiScript gDNA Removal RT MasterMix; Kangwei Century Biotechnology Co., Ltd., Taizhou, China), with all operations performed according to the manufacturer’s instructions.

### 4.7. Quantitative Real-Time PCR (qRT-PCR)

The primers listed in this study (Table 1) were designed using Primer 5.0 software. Gene expression levels were determined by real-time quantitative PCR (LightCycler 480 Multiwell Plate 96; Roche, Indianapolis, IN, USA) using the TaKaRa TB Green^®^ Premix Ex Taq™ II (Tli RNaseH Plus) kit, with *GAPDH* as the internal reference gene. Each reaction consisted of a 10 µL system containing cDNA (1 µL), RNase-free water (3 µL), forward and reverse primers (0.5 µL each), and TB Green^®^ Premix Ex Taq™ II (5 µL).

### 4.8. CCK-8 Method for Detecting Cell Proliferation

Passage the cells into a 96-well plate, overnight culture, transfection treatment, 6 replicates per treatment, and place them in a 37 °C, 5% CO_2_ incubator. Add 90 μL of 10% complete culture medium and 10 μL of CCK-8 to each well, taking care to avoid the formation of bubbles. Wrap the culture plate with tin foil and incubate it in a dark incubator. After 4 h, take out the culture plate and detect the OD_450 nm_ value in an enzyme-linked immunosorbent assay (ELISA) reader.

### 4.9. Cell Cycle Assay Middling

Forty-eight hours after transfection with si-INSL3 and NC, granulosa cells from *Dabie Mountain cattle* were digested with 0.25% trypsin (37 °C, 3 min) and centrifuged (4000 rpm, 5 min). After washing with 70% ethanol and overnight incubation at 4 °C, collected cells were stained with RNase A (100 µL) and PI solution (400 µL) for 30 min. Cell cycle progression was assessed using FACSVerse Calibur flow cytometer (BD Biosciences, San Jose, CA, USA). The proportion distribution of each cell cycle phase in three independent experiments was analyzed using ModFit v 6.0 software.

### 4.10. Statistical Analysis

Data indicated as mean ± SEM were obtained from three independent replicates at least. The statistically significant difference between groups was determined using paired-samples *t*-test in Graph-Pad. The cut-off value adjusted to a statistically significant difference was *p* < 0.05.

## 5. Conclusions

To investigate the molecular mechanisms regulating *Dabie Mountain cattle* follicular development, this study collected small, medium, and big follicles for transcriptome sequencing. A total of 19 genes were identified as upregulated and 227 as downregulated in the comparisons B vs. S, B vs. M, and M vs. S. The results revealed that *INSL3* gene expression was highest in small follicles and was almost absent in big follicles. Subsequent in vitro studies on *INSL3* revealed that knocking down *INSL3* expression significantly promoted GCs proliferation by markedly upregulating the expression of proliferation-related genes *Myc*, *PCNA*, *Cytochrome C*, and *Cyclin D1*. This treatment also significantly reduced the number of G1-phase cells and markedly increased the number of S-phase cells. In conclusion, *INSL3* influences follicular development in *Dabie Mountain cattle* by regulating GCs proliferation, thereby contributing to the regulation of their reproductive efficiency.

## Figures and Tables

**Table 1:** Primer sequences for quantitative real-time PCR and si-INSL3 sequences.

Gene Name	Primer Sequences
si-INSL3(239)	F: GUGGCUGGAAGGACAACAUTT R: AUGUUGUCCUUCCAGCCACTT
si-INSL3(392)	F: CUGCACCCGGCAAGACCUGTT R: CAGGUCUUGCCGGGUGCAGTT
siNC	F: UUCUCCGAACGUGUCACGUTT R: ACGUGACACGUUCGGAGAATT
*GAPDH*	F: GTGCCCAGTGCCATAC R: CCATCAGCGTCTCCTC
*Myc*	F: CCCACCCACGACCAGTA R: GCTGTGAGGAGGTTTGC
*PCNA*	F: AGAAAGTGCTGGAGGC R: TCGGAGCGAAGGGTTA
*Cyclin D1*	F: CTGGTCCTGGTGAACAAA R: TGGCACAGAGGGCAAC
*Cytochrome C*	F: GTGCCCAGTGCCATAC R: CCATCAGCGTCTCCTC

## Data Availability

The data presented in this study are available on request from the corresponding author due to (The experimental data is kept by the corresponding author due to privacy concerns).
